# Quercetin mitigates size-dependent oxidative and metabolic toxicity of citrate-coated silver nanoparticles in human erythrocytes

**DOI:** 10.1007/s00204-026-04308-z

**Published:** 2026-02-17

**Authors:** Inês Santos, Vera M. Costa, Félix Carvalho, Eduarda Fernandes, Marisa Freitas

**Affiliations:** 1 LAQV, REQUIMTE, Laboratory of Applied Chemistry, Department of Chemical Sciences, Faculty of Pharmacy, University of Porto, Rua de Jorge Viterbo Ferreira n.º 228, 4050-313 Porto, Portugal; 2 Laboratory of Toxicology, UCIBIO - Applied Molecular Biosciences Unit, Department of Biological Sciences, Faculty of Pharmacy, University of Porto, 4050-313 Porto, Portugal; 3 Associate Laboratory i4HB - Institute for Health and Bioeconomy, Faculty of Pharmacy, University of Porto, 4050-313 Porto, Portugal; 4 Faculty of Medicine, University of Porto (FMUP), Rua Alameda Prof Hernâni Monteiro, 4200-319 Porto, Portugal

**Keywords:** Silver nanoparticles, Quercetin, Erythrocytes, Toxicity

## Abstract

Silver nanoparticles (AgNP) are extensively used in consumer and medical products, making systemic exposure increasingly plausible. Once in circulation, AgNP directly interact with erythrocytes; however, AgNP effects on human erythrocytes remain poorly characterised, despite the central role of erythrocytes in maintaining blood homeostasis and the likelihood of direct nanoparticle contact following human exposure. Quercetin, a dietary flavonoid with potent antioxidant and anti-inflammatory activities, has shown cytoprotective potential against oxidative damage in various cell types, but its ability to counteract AgNP-induced erythrotoxicity has not yet been established. Accordingly, we (i) evaluated the effects of citrate-coated AgNP of 5, 10, and 50 nm on human erythrocytes and (ii) investigated the potential protective role of quercetin against AgNP-induced toxicity. The results showed that all citrate-coated AgNP induced haemolysis in human erythrocytes. Exposure to all three nanoparticle sizes markedly increased intracellular levels of pro-oxidant reactive species (RS). In particular, 10 and 50 nm AgNP caused a significant decrease in total glutathione (GSH) levels. Additionally, all tested AgNP also induced ATP decrease. Moreover, the 5 and 10 nm AgNP increased intracellular calcium levels. Remarkably, pre-treatment with quercetin conferred protection against almost all tested cytotoxic endpoints. However, quercetin failed to prevent total GSH loss and even exacerbated ATP depletion. Collectively, these findings provide the first integrated evidence of three sizes of AgNP-induced toxicity in human erythrocytes, demonstrating that quercetin confers broad, though not universal, protection. This underscores its potential as a modulator of nanoparticle-induced oxidative and metabolic damage.

**Supplementary Information:**

The online version contains supplementary material available at 10.1007/s00204-026-04308-z.

## Introduction

Silver nanoparticles (AgNP) have emerged as one of the most widely used classes of nanoparticles (NP), largely due to their distinctive physicochemical characteristics (Abbasi et al. 2016; Almatroudi 2020). Furthermore, due to their remarkable antimicrobial properties (Bruna et al. 2021; Zhang et al. 2016), they have attracted considerable attention and are increasingly incorporated into diverse applications, including textiles, food packaging, and medical products (Nie et al. 2023). However, the growing prevalence of AgNP in consumer products, coupled with the likelihood of continuous human exposure, has raised significant concerns regarding their potential adverse health effects. Current evidence indicates that AgNP-induced toxicity is mediated by multiple interrrelated mechanisms, including, oxidative stress (OS), inflammation, mitochondrial dysfunction, endoplasmic reticulum stress, DNA damage, and ultimately, cell death (Santos et al. 2025).

AgNP can enter the human body via ingestion, inhalation, or dermal absorption, and are capable of reaching the circulatory system regardless of the route of entry. Once in the bloodstream, they interact with various blood components, namely erythrocytes, which are the most abundant circulating blood cells. Consequently, a comprehensive assessment of the haemocompatibility of AgNP is essential, considering the possible interactions with blood components and, ultimately, the cardiovascular system. In this context, erythrocytes serve as a primary cellular model for evaluating nanotoxicity (Chen et al. 2015; Ferdous and Nemmar 2020). Despite this, the hemotoxic potential of AgNP remains insufficiently understood. The limited studies available suggest that exposure to AgNP may result in haemolysis, OS, and eryptosis, a type of programmed cell death (Dreischer et al. 2022; Ferdous et al. 2018; Bian et al. 2019; Huang et al. 2016). This process is characterised by membrane blebbing, which is calpain-mediated, cell membrane scrambling of phosphatidylserine, and cell shrinkage, which are triggered by several mechanisms, including calcium entry through Ca^2+^-permeable channels, caspases, ceramide, and deranged activity of several kinases, such as protein kinase C (PKC). These processes can be activated by hyperosmotic shock, energy depletion and OS (Dreischer et al. 2022; Pretorius et al. 2016; Tkachenko and Havránek 2023). Nevertheless, the precise mechanisms underlying AgNP-induced erythrocyte toxicity remain not completely understood.

The potential harmful effects associated with AgNP exposure underscore the urgent need to identify compounds that can mitigate their toxicity. In this context, quercetin (3,3′,4′,5,7-pentahydroxyflavone) stands out as a major dietary flavonoid. It is widely found in fruits and vegetables, with extensive documentation of its broad spectrum of biological activities, particularly its potent antioxidant properties (Aghababaei and Hadidi 2023; Anand David et al. 2016; Carrillo-Martinez et al. 2024). Furthermore, some studies have recognised quercetin as one of the most effective flavonoids in mitigating the adverse cellular effects elicited by AgNP exposure (Santos et al. 2025; Sousa et al. 2025b). However, its protective role against AgNP-induced toxicity in human erythrocytes has not yet been described.

Accordingly, the present study was designed with two main objectives: (a) to evaluate the effect of various concentrations of citrate-coated AgNP of three sizes (5, 10 and 50 nm) on human erythrocytes, and (b) to investigate the protective role of quercetin in AgNP-treated cells.

## Materials and methods

### Materials

BioPure citrate-coated AgNP (5, 10 and 50 nm) and citrate were obtained from nanoComposix (San Diego, CA). 5,5′-Dithiobis(2-nitrobenzoic acid) (DTNB), ATP, bovine serum albumin (BSA), calcium ionophore A23187, 2′,7′-dichlorodihydrofluorescein diacetate (DCFH-DA), dimethylsulfoxide (DMSO), Dulbecco’s phosphate buffered saline (DPBS) without calcium chloride and magnesium chloride, Gö6983, l-glutathione reduced (GSH), glutathione reductase (GR) from baker’s yeast (*S. cerevisiae*), *tert*-butyl hydroperoxide (tBOOH) solution (Luperox^®^), luciferase from *Photinus pyralis* (firefly), 70% (w/v) perchloric acid (HClO_4_), quercetin (≥ 98%, CAS 117-39-5), silver nitrate (AgNO_3_), sodium fluoride (NaF), trypan blue solution 0.4% and Triton™ X-100 were obtained from Sigma Chemical Co (St. Louis, Mo, USA). Fluo-4 AM was obtained from Life Technologies, Thermo Fisher Scientific, Inc (Waltham, MA, USA). Nicotinamide adenine dinucleotide phosphate (NADPH) tetrasodium salt was obtained from PanReac AppliChem, ITW Reagents (Darmstadt, Germany); D-Luciferin sodium salt was obtained from Abcam (Cambridge, UK). DC™ Protein Assay kit was obtained from BioRad Laboratories (Hercules, CA, USA).

### Methods

#### AgNP characterisation

The citrate-coated AgNP of the three sizes, 5, 10 and 50 nm citrate-coated were characterised in an aqueous medium and the results were kindly provided by the manufacturer. Each NP was characterised using transmission electron microscopy (TEM) to determine the diameter and shape distributions, UV–visible spectroscopy to measure the optical properties, dynamic light scattering to determine particle hydrodynamic diameter, and zeta potential measurement to determine particle surface charge. BioPure nanoparticles were extensively washed with the suspending solvent to remove residual reactants from the manufacturing process. Mass concentration was determined by inductively coupled plasma mass spectroscopy (ICP-MS). The particles were sterile-filtered using filters with a 0.22 μm pore size and tested for endotoxin contamination before delivery. Throughout the study, nanomaterials were stored at 4 °C.

**Fig. 1:**
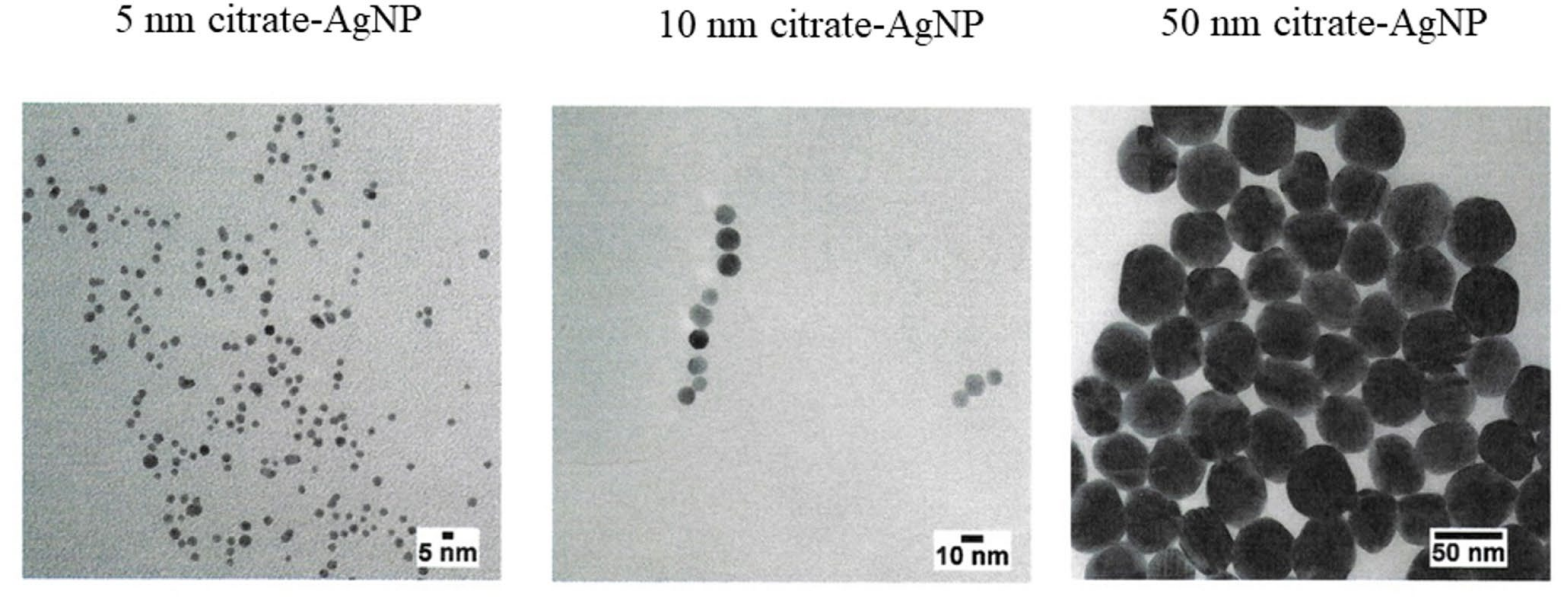
TEM characterisation of, **a** 5 nm, **b** 10 nm and **c** 50 nm citrate-coated AgNP. [images kindly provided by the manufacturer, nanoComposix (San Diego, CA)]

The citrate-coated AgNP (5, 10, and 50 nm) were characterised using TEM by the manufacturer (nanoComposix). According to the data, in aqueous medium, the 5, 10, and 50 nm citrate-coated AgNP were spherical in shape and the average individual sizes were 4.1 ± 0.7 nm, 9.8 ± 1.7 nm and 49 ± 5 nm, respectively (Fig. 1; Table 1). Furthermore, the zeta potentials of the 10 and 50 nm citrate-coated AgNP were − 34 mV and − 51 mV, respectively, with corresponding hydrodynamic diameters of 14 nm and 52 nm, respectively. However, for the 5 nm citrate-coated AgNP, the manufacturer did not provide these data, indicating that the particle concentration was too low to generate sufficient scattered light for accurate measurement.

**Table 1:** Diameter measured by TEM of the three sizes of citrate-coated AgNP (5, 10 and 50 nm), according to the manufacturer, nanoComposix (San Diego, CA)

	Citrate-coated AgNP		
	5 nm	10 nm	50 nm
Diameter [Size mean ± SD (nm)]	4.1 ± 0.7 nm	9.8 ± 1.7 nm	49 ± 5 nm
Coefficient of Variation (%)	17%	17.5%	10.4%

#### Isolation of erythrocytes

All patient-related procedures and protocols were performed in accordance with the Declaration of Helsinki and approved by the Ethics Committee of Centro Hospitalar do Porto. Following written informed consent, venous blood was drawn by antecubital venipuncture into K_3_EDTA vacuum tubes at the blood bank of Centro Hospitalar do Porto-Hospital de Santo António. Erythrocytes were isolated based on the procedure described by Chisté et al. (2014), with slight modifications. The collected blood samples were transferred to sterile conical tubes containing 6 mL of PBS. The tubes were then centrifuged at 1500 × *g* for 5 min at 4 °C. The supernatant was discarded, and the erythrocyte pellet was resuspended in 6 mL of PBS. The resuspended pellet was then subjected to a second centrifugation at 1500 x g for 5 min at 4 °C. This process was repeated three times. After the third centrifugation, the supernatant was discarded, and the pellet was resuspended in a 5.5 mM Tris-glucose buffer [1.26 mM CaCl_2_, 5.37 mM KCl, 0.81 mM MgSO_4_, 140 mM NaCl, 25 mM Tris (trizma), 5.5 mM D-glucose, pH 7.4]. Subsequently, a dilution (1:800) of the cell suspension was prepared, and cell counting and viability was performed using the trypan blue exclusion method with a Neubauer chamber (Chisté et al. 2014).

#### Haemolysis assay

This assay evaluates AgNP-induced damage by quantifying haemoglobin released into the surrounding medium, measured at 540 nm, following the procedure previously described (Chisté et al. 2014). A 200 µL volume of the cell suspension with a final density of 30 × 10^6^ cells/mL was seeded into a 48-well plate and pre-treated with quercetin (10, 25, or 50 µM) or DMSO for 30 min at 37 °C, under gentle shaking, protected from light. Following this pre-incubation step (with or without quercetin), 5, 10 and 50 nm citrate-coated AgNP at different concentrations (10, 25, 50 µg/mL) were tested. Moreover, Triton X-100 (0.1%) (positive control for haemolysis) (Huang et al. 2016), and citrate (coating) (0.02 µg/mL) or AgNO_3_ (0.5 µg/mL) [corresponding to the levels present in the AgNP suspensions at their highest concentration (50 µg/mL)] were used. All the aforementioned conditions were incubated for 5 h, at 37 °C, with gentle shaking, protected from light. At the end of the incubation period, the content of the wells was collected and centrifuged at 1500 × *g* for 5 min at 4 °C. Subsequently, the supernatant was transferred to a 96-well plate, and the absorbance was measured at 540 nm in a microplate reader (Cytation 5 imaging reader, Biotek Instruments, Inc.).

#### Assessment of reactive species production

DCFH-DA is a non-fluorescent compound that can permeate through the cell membrane. Once inside the cell, it is hydrolysed by intracellular esterases to generate 2′,7′-dichlorodihydrofluorescein (DCFH). In the presence of reactive species (RS), such as H_2_O_2_, ^•^OH or ONOO^−^. DCFH is oxidised to form the highly fluorescent compound 2′,7′-dichlorofluorescein (DCF) (Soares et al. 2019). Following isolation, 190 μL of erythrocytes (final cellular density of 5 × 10⁶ cells/mL) were incubated with 5 µM DCFH-DA in a black 96-well plate at 37 °C for 30 min under gentle shaking, protected from light. Following this incubation, quercetin or DMSO was added at concentrations of 0.5, 1, and 5 µM and incubated for an additional 30 min under the same conditions. Subsequently, the citrate-coated AgNP (10, 25, and 50 µg/mL) with or without quercetin, tBOOH (20 µM, being used as a positive control) (Besedina et al. 2022), citrate (0.02 µg/mL) or AgNO_3_ (0.5 µg/mL) were added. The fluorescence intensity was then measured using a microplate reader (Synergy HT reader, Biotek instruments, INC), over a period of 3 h, using excitation and emission wavelengths of 485 and 528 nm, respectively. The slope of the fluorescence curve between 2 and 3 h was used for analysis. Interference tests were performed and showed that neither AgNP nor quercetin affected the assay performance.

#### Quantification of total glutathione

Total glutathione in the erythrocyte suspension was quantified by the DTNB-GSH reductase recycling assay, as previously described by (Barbosa et al. 2012; Chisté et al. 2014), with minor modifications. A volume of 250 µL of erythrocytes (final cellular density of 5 × 10^6^ cells/mL) were seeded into a 96-well plate and pre-treated with quercetin (10 and 50 µM) or DMSO for 30 min, at 37 °C under gentle shaking, protected from light. Subsequently, cells were incubated with tBOOH (250 µM) as a positive control for 30 min (Lapshina et al. 2005; Besedina et al. 2022) or the three sizes (5, 10, or 50 nm) of citrate‑coated AgNP (10, 25 and 50 µg/mL) with or without quercetin, AgNO_3_ (0.5 µg/mL) or citrate (0.02 µg/mL) was added, and the plate was incubated for an additional 45 min, under the same conditions as previously described. After treatment, the cells were transferred to conical microtubes and centrifuged at 1500 × *g* for 5 min at 4 °C. The supernatants were discarded, and the pellets were resuspended in 5% (w/v) HClO_4_, followed by a centrifugation at 16,000 × *g* for 10 min at 4 °C. The supernatants were collected for total GSH determinations and stored at − 80 °C until use. The pellet was used for protein determination and stored at − 20 °C.

For the quantification of total GSH, standard solutions of GSH were prepared, ranging from 0 to 15 µM. Each sample was neutralised with ice-cold 0.76 M KHCO_3_ and centrifuged at 16,000 × *g* for 2 min at 4 °C. Subsequently, the supernatant was transferred to a 96‑well plate and mixed with the reagent solution (1.3 mM DTNB, 0.22 mM NADPH). Both the reagent solution and enzymatic solution of glutathione reductase (10 U/mL) were prepared in phosphate buffer (71.5 mM Na_2_HPO_4_; 71.5 mM NaH_2_PO_4_; 0.63 mM EDTA, pH 7.5). After 15 min of incubation at 30 °C in a microplate reader, glutathione reductase was added, and the absorbance was monitored every 10 s for 3 min at 415 nm (PowerWave X, Biotek Instruments, Inc.).

#### Measurement of ATP levels

Intracellular ATP levels were determined by a bioluminescence-based method using luciferase, which catalyses the ATP-dependent oxidation of luciferin, as previously described by Rodrigues et al. (2020), with modifications. Sample preparation followed the procedure mentioned for total GSH quantification. In detail, 250 µl of the isolated erythrocytes were first transferred to a 96-well plate. The cells were pre-incubated with quercetin or DMSO to each well to achieve final concentrations of 10 and 50 µM. The plate was gently agitated at 37 °C for 30 min in the dark. After this incubation period, cells were exposed (with or without quercetin) to the different sizes of citrate-coated AgNP (5, 10, or 50 nm) at a final concentration of 10, 25, or 50 µg/mL, the positive control NaF (200 µM) (Agalakova and Gusev 2012), AgNO_3_ (0.5 µg/mL) or citrate (0.02 µg/mL), for 45 min, under the same conditions as previously described. Following this incubation, the cells were collected and centrifuged at 1,500 × *g* for 5 min at 4 °C. The remaining pellets were resuspended in 5% (w/v) HClO_4_ to release intracellular ATP. Then, a centrifugation at 16,000 × *g* for 10 min at 4 °C followed. The supernatant was collected and analysed immediately for ATP determinations. The pellets were used for protein determination.

For ATP quantification, standard solutions were prepared, ranging from 0 to 10 µM. A stock solution of D-luciferin (90.9 mg/L) and luciferase derived from *Photinus pyralis* (firefly), at a final enzymatic activity of 3,000,000 U/mL, was prepared in luciferin–luciferase reaction buffer (50 mM glycine, 10 mM MgSO_4_, 1 mM Tris, 0.55 mM EDTA, 0.1% BSA, pH 7.6). The reagents were aliquoted in light-protected tubes and stored at − 80 °C until use. All samples and standards were neutralised using ice-cold 0.76 M KHCO_3_, and then the mixture was thoroughly vortexed and centrifuged at 16,000 × *g* for 1 min at 4 °C. The resulting supernatants were transferred into a white 96-well plate. Then the luciferin-luciferase reagent was added and the emitted bioluminescence was measured immediately in the microplate reader (Synergy HT reader, Biotek Instruments, Inc.). All measurements were performed in duplicate.

#### Protein determination

The protein pellets mentioned above were resuspended in 0.4 M NaOH and incubated at 60 °C for 1 h. The total protein levels were assessed using the DC™ Bio-Rad Protein Assay Kit, using BSA as a standard.

#### Measurement of intracellular calcium levels

The calcium-sensitive probe Fluo-4 AM readily permeates cell membranes and is hydrolysed by intracellular esterases. Cleavage of its acetoxymethyl (AM) ester group generates the active Fluo-4 form, which remains sequestered within the cytosol (Goldshmidt and Michaeli 2011). This method was implemented based on the procedure described by (Ribeiro et al. 2018), with modifications. A 2 mL volume of isolated erythrocytes (final cellular density of 10 × 10^6^ cells/mL) in PBS, were incubated with 2 µM of Fluo-4 AM probe in a 12-well plate for 1 h at 37 °C with gentle agitation and protected from light. Subsequently, the cells were subjected to centrifugation at 1500 x *g* for 5 min at 20 °C and washed twice with PBS. After the final wash, the cells were resuspended in Tris-glucose buffer. Thereafter, 220 µL of the resuspended cells were first transferred to a black 96-well plate, and quercetin or DMSO was added to each well to achieve final concentrations of 10, 25, and 50 µM. The plate was incubated for 30 min at 37 °C under gentle agitation and protected from light. After this incubation, the 5, 10 and 50 nm citrate-coated AgNP (10, 25 and 50 µg/mL) with or without quercetin, ionophore A23187 (5 µM) used as a positive control (Kuck et al. 2020; Bian et al. 2019), the coating per se (citrate) (0.02 µg/mL) or AgNO_3_ (0.5 µg/mL) were added to the plate. The calcium levels were monitored for 1 h and 30 min. The fluorescence intensity was then measured using excitation and emission wavelengths of 485 and 528 nm, respectively (Cytation 5 imaging reader, Biotek Instruments, Inc.). For the ionophore, the slope of the fluorescence curve during the first 10 min was used, whereas for the AgNP, the slope between 1 h and 1 h 20 min was analysed.

##### Involvement of protein kinase C in the increase of calcium levels in erythrocytes

The effect of PKC inhibition on AgNP-induced calcium increase was assessed using Gö6983, a PKC inhibitor, following the same protocol as mencioned above. In detail, after the Fluo-4 AM loading and the subsequent washes, the resuspended erythrocytes were transferred to a black 96-well plate, followed by the addition of Gö6983, at a final concentration of 1 µM or 2.5 µM. The cells were incubated with the PKC inhibitor for 15 min at 37 °C under gentle agitation and protected from light. Following this treatment, the cells were exposed to 5 and 10 nm citrate-coated AgNP at a concentration of 50 µg/mL, which corresponded to the condition that elicited the most pronounced increase in intracellular calcium levels. The measurement of calcium release was performed as described above.

### Statistical analysis

Statistical analysis was conducted using GraphPad Prism software (version 9.0, CA, USA). Data are expressed as the average ± SEM from a minimum of three independent experiments, which were analysed. Comparisons among experimental conditions were performed using one-way analysis of variance (ANOVA), followed by Bonferroni’s *post hoc* test. A *p*-value < 0.05 was considered statistically significant.

## Results

### Haemolysis

**Fig. 2:**
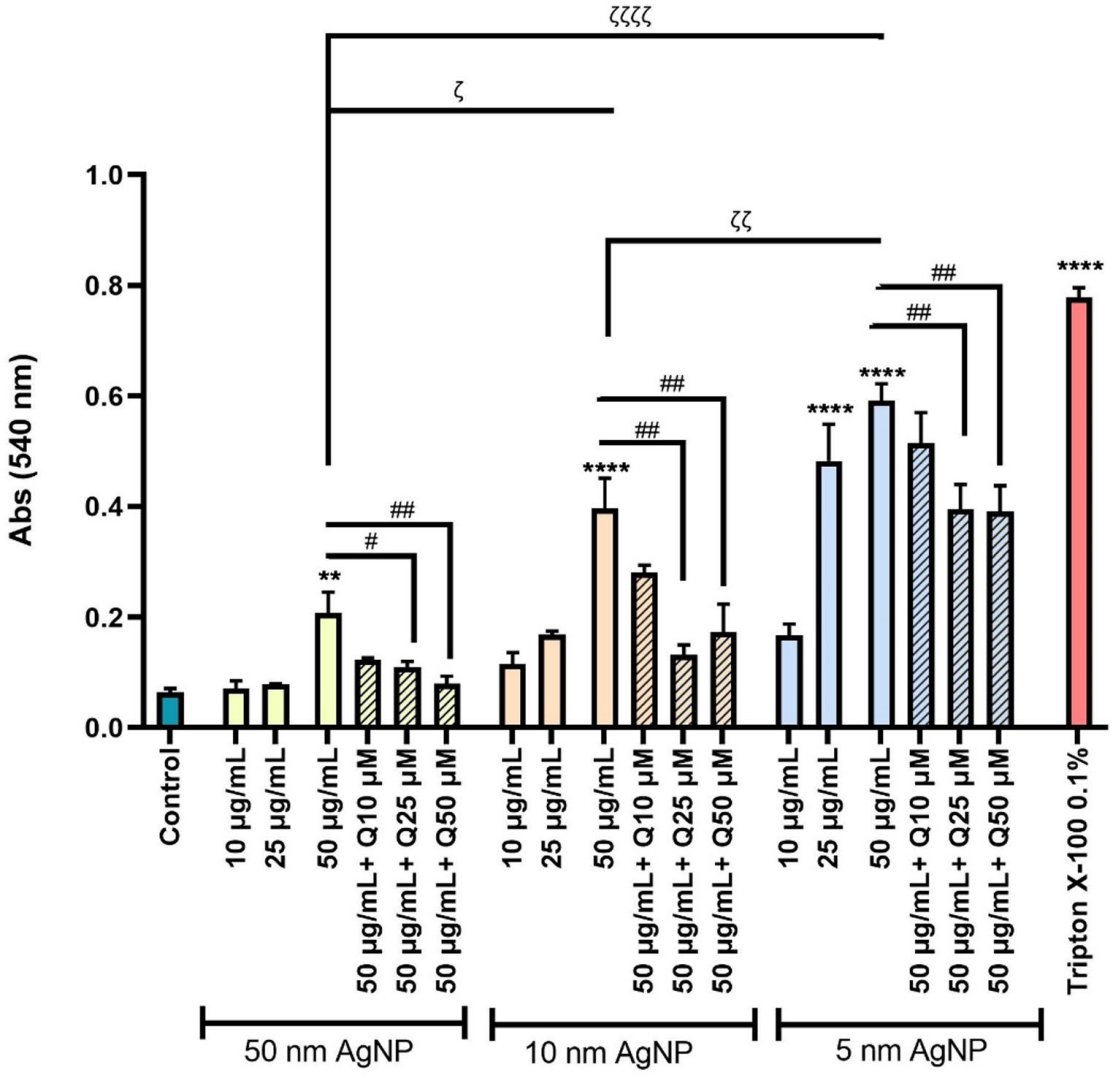
Haemolytic effect of 5, 10 and 50 nm citrate-coated AgNP (10–50 µg/mL) and Triton X-100 (0.1%) on erythrocytes after 5 h of exposure and the modulatory effect of quercetin (10–50 µM). **p < 0.01, ****p < 0.0001, compared to control (untreated cells), ^ζ^ *p* < 0.05, ^ζ ζ^ *p* < 0.01, ^ζ ζ ζ ζ^ *p* < 0.0001, as indicated by the connecting lines and ^#^ *p* < 0.05, ^# #^ *p* < 0.01, compared to citrate-coated AgNP, respectively. Each value represents the mean ± SEM of at least three independent assays

Figure 2 shows that citrate-coated AgNP of all sizes significantly increased haemolysis after 5 h of exposure. A size-dependent effect was observed, with the smallest citrate-coated AgNP (5 nm) inducing the highest haemolytic effect, followed by the 10 nm, and finally the 50 nm particles. Considering the observation that 5, 10 and 50 nm citrate-coated AgNP, at their maximum concentration (50 µg/mL), after 5 h of exposure, elicited significant haemolysis, this was the condition selected to evaluate the protective effect of quercetin. In this context, erythrocytes were initially incubated with quercetin (10, 25, and 50 µM) and subsequently exposed to citrate-coated AgNP (50 µg/mL) 30 min later. The results obtained demonstrate that quercetin exerts a protective effect, significantly reducing the haemolysis induced by the three sizes of citrate-coated AgNP, even at 25 µM. The citrate coating, Ag⁺ or quercetin tested individually did not induce any significant changes in haemolysis (Fig. S1). Triton X-100 (0.1%) was used as a positive control.

### Reactive species production

**Fig. 3:**
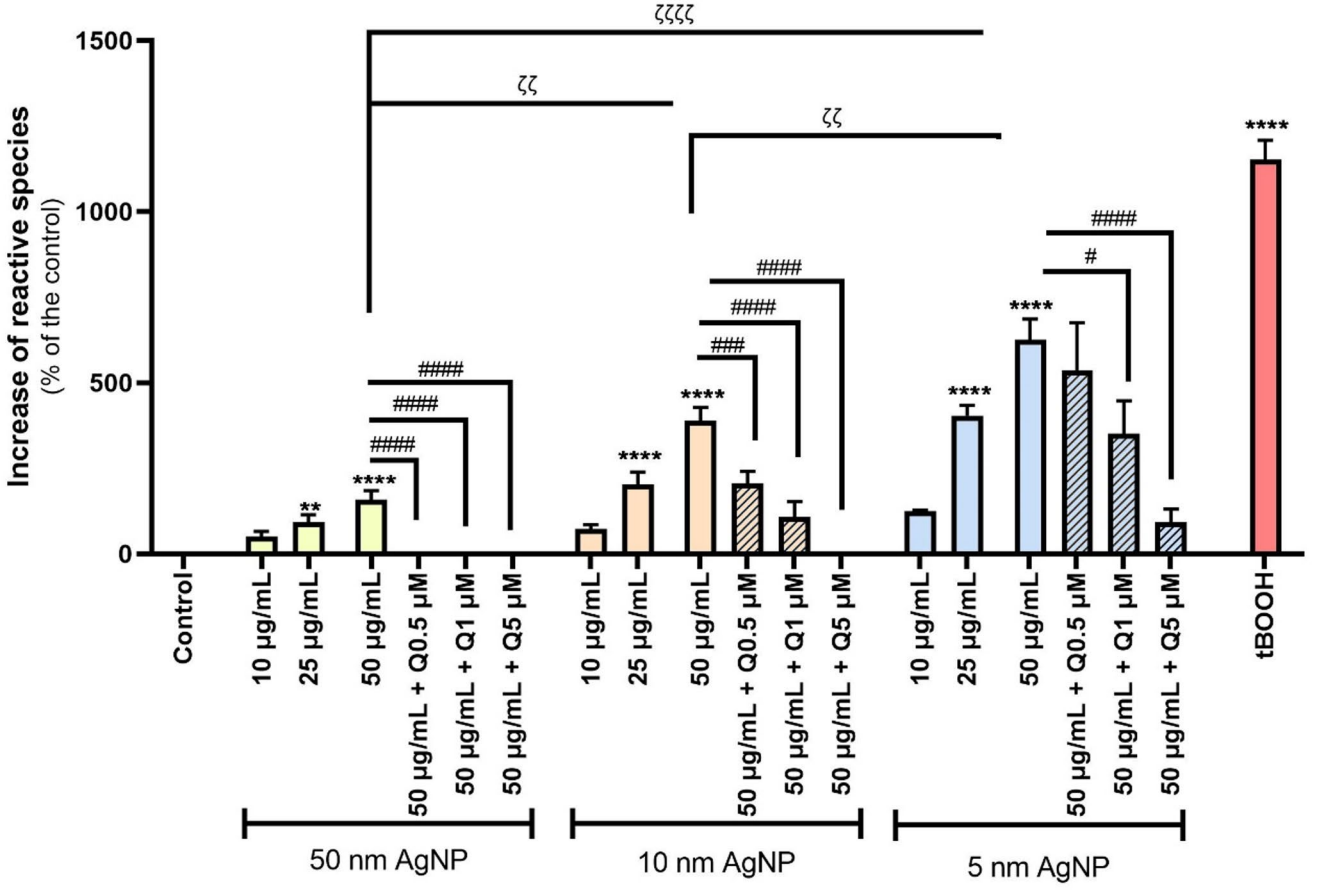
Reactive species formation by erythrocytes, after exposure to 5, 10 and 50 nm citrate-coated AgNP (10–50 µg/mL) and tBOOH (20 µM), and the modulatory effect of quercetin (0.5-5.0 µM). **p < 0.01, ****p < 0.0001, compared to control (untreated cells), ^ζ ζ^ *p* < 0.01, ^ζ ζ ζ ζ^ *p* < 0.0001, as indicated by the connecting lines and ^#^ *p* < 0.05, ^###^ *p* < 0.001, ^####^ *p* < 0.0001, compared to citrate-coated AgNP, respectively. Each value represents the mean ± SEM of at least three independent assays

The results presented in Fig. 3 demonstrate that citrate-coated AgNP of all three sizes, at concentrations of 25 and 50 µg/mL, significantly increase RS levels on human erythrocytes. Among the three sizes of AgNP tested, the 5 nm citrate-coated AgNP induced the production of the highest levels of RS. This increase was significantly greater than that observed with 10 and 50 nm AgNP, indicating a clear size-dependent effect on RS production. Based on these results, erythrocytes were pre-incubated with quercetin at concentrations of 0.5, 1.0, and 5.0 µM, followed by the exposure to citrate-coated AgNP (50 µg/mL). The results obtained revealed that quercetin, even at low concentrations, significantly mitigate the increase of RS induced by AgNP. Notably, even at the lowest tested concentration (0.5 µM), quercetin was still effective in counteracting the production of RS induced by the 10 and 50 nm AgNP. In contrast, this concentration of quercetin was insufficient to significantly reduce the RS levels in erythrocytes exposed to 5 nm AgNP. The effect of Ag^+^, citrate coating or quercetin alone was evaluated and no significant alterations in RS levels were detected (Fig. S2). The tBOOH (20 µM) was used as a positive control.

### Total glutathione levels

**Fig. 4:**
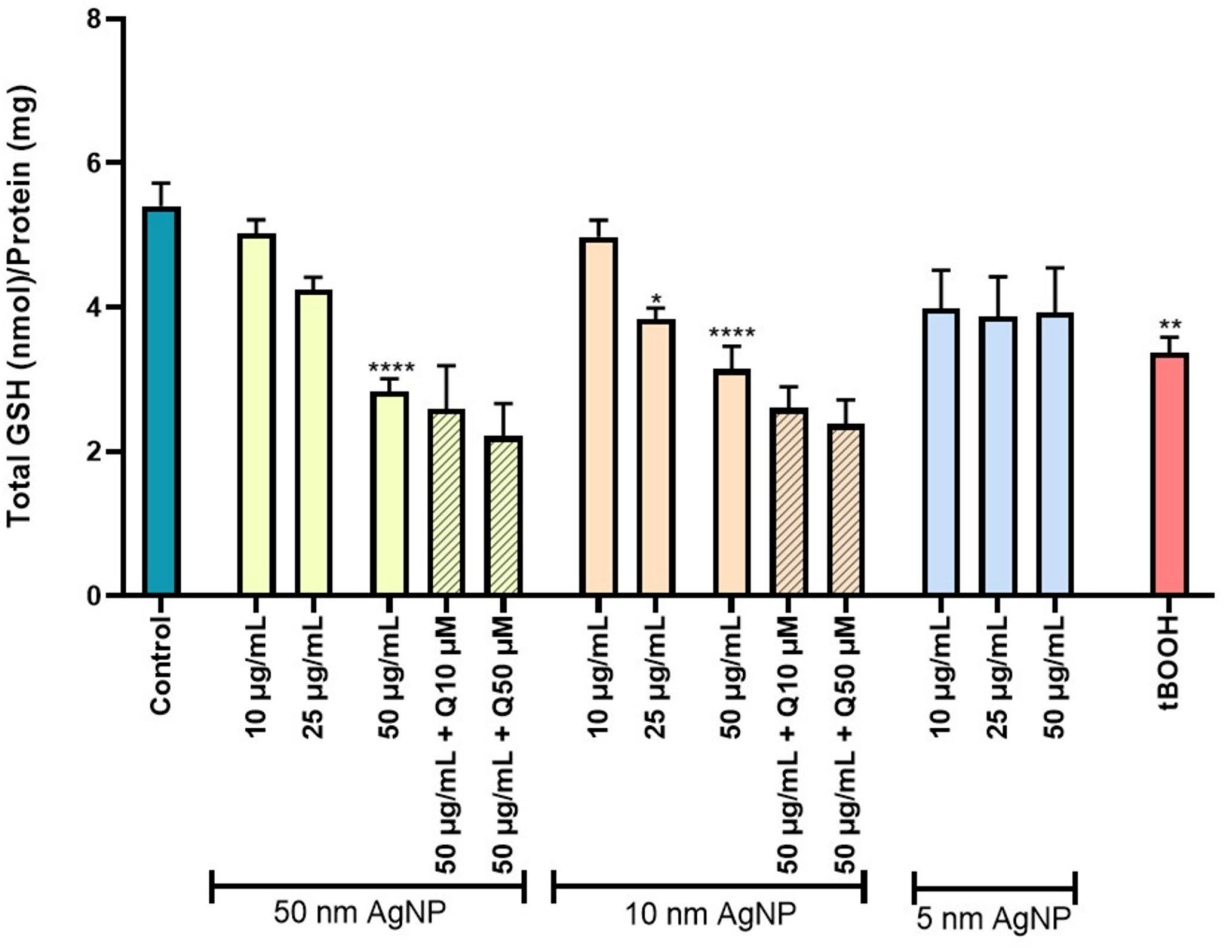
Total GSH levels on erythrocytes, after exposure to 5, 10 and 50 nm citrate-coated AgNP (10–50 µg/mL) and tBOOH (250 µM), and the modulatory effect of quercetin (10–50 µM). *p < 0.05, **p < 0.01 ****p < 0.0001, compared to control (untreated cells). Each value represents the mean ± SEM of at least three independent assays

The data presented in Fig. 4 indicate that exposure to citrate-coated AgNP leads to depletion of total GSH. Specifically, exposure to 10 and 50 nm of citrate-coated AgNP (50 µg/mL) resulted in a significant reduction in total GSH levels. Additionally, the 10 nm citrate-coated AgNP also induced a significant decrease at 25 µg/mL. Interestingly, the exposure to 5 nm citrate-coated AgNP, regardless of concentration, did not significantly alter the total GSH levels compared to the control (untreated cells). Considering these results, the effect of quercetin was evaluated following exposure to 10 and 50 nm citrate-coated AgNP at a concentration of 50 µg/mL. Quercetin pre-treatment was ineffective in preventing NP-induced alterations in erythrocyte total GSH. Furthermore, neither Ag⁺, citrate or the quercetin per se induced significant changes in total GSH content (Fig. S3). The tBOOH (250 µM) was used as a positive control to decrease total GSH levels.

### ATP levels

**Fig. 5:**
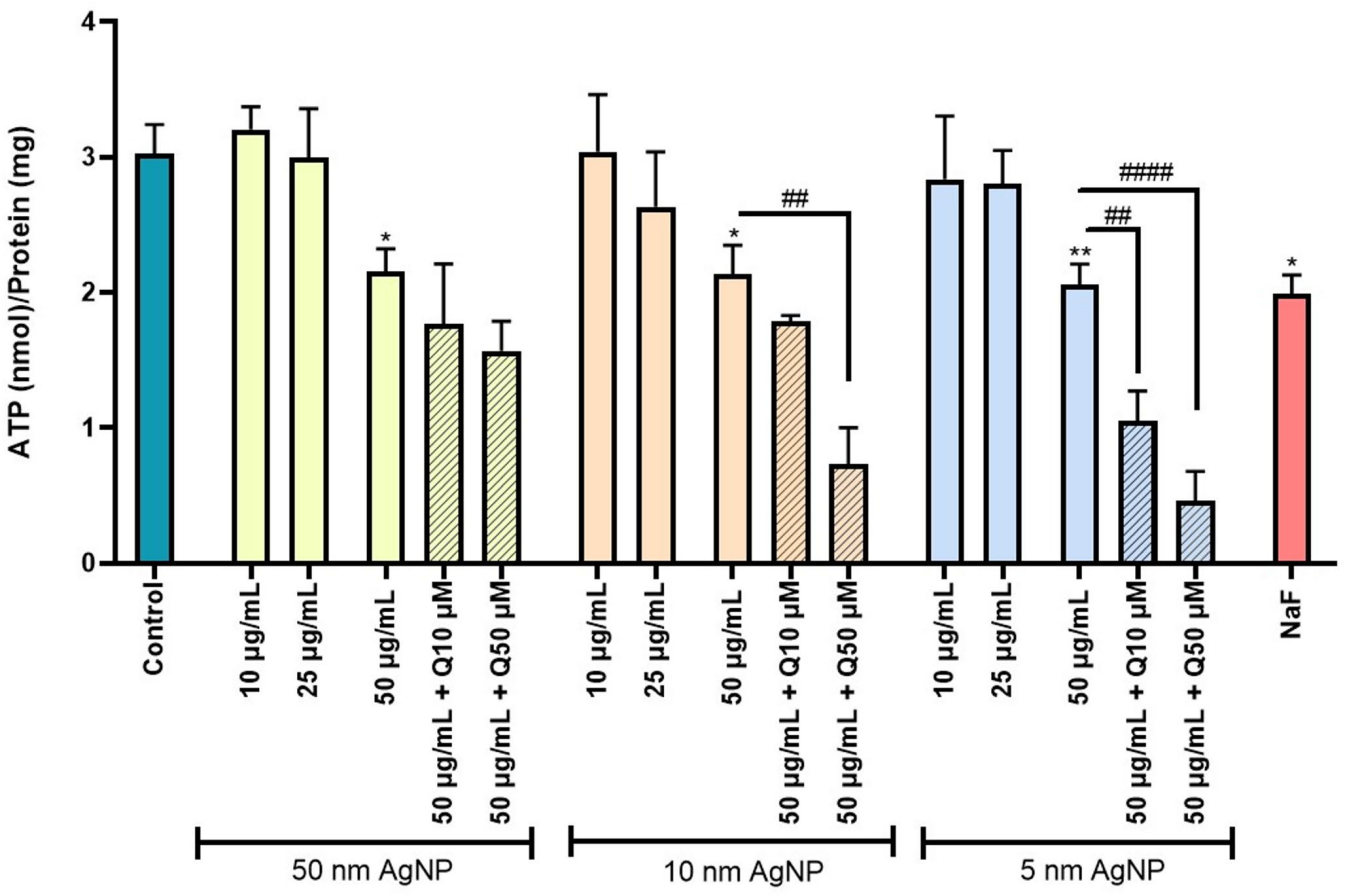
ATP levels on erythrocytes after exposure to 5, 10 and 50 nm citrate-coated AgNP (10–50 µg/mL) and NaF (200 µM), and the modulatory effect of quercetin (10–50 µM). *p < 0.05, **p < 0.01, compared to control (untreated cells), ^##^ *p* < 0.01, ^####^ *p* < 0.0001, compared to citrate-coated AgNP, respectively. Each value represents the mean ± SEM of at least three independent assays

The results presented in Fig. 5 demonstrate that all three sizes of the citrate-coated AgNP, at the highest tested concentration (50 µg/mL) induced a significant decrease in ATP levels. Considering the observed results, the potential of quercetin as a protective agent on ATP level reduction was evaluated. Interestingly, for the 5 and 10 nm citrate-coated AgNP, the presence of quercetin (50 µM) resulted in an even greater reduction in ATP levels. Notably, for the smallest AgNP (5 nm), this effect was already evident even at a lower quercetin concentration (10 µM). On the other hand, for the largest AgNP size tested (50 nm), quercetin did not cause any significant change in ATP levels. Furthermore, citrate, Ag^+^ and quercetin per se did not demonstrate any significant alterations in ATP levels compared to the control (untreated cells) (Fig. S4). The NaF (200 µM) was used as a positive control to decrease ATP level.

### Intracellular calcium levels

**Fig. 6:**
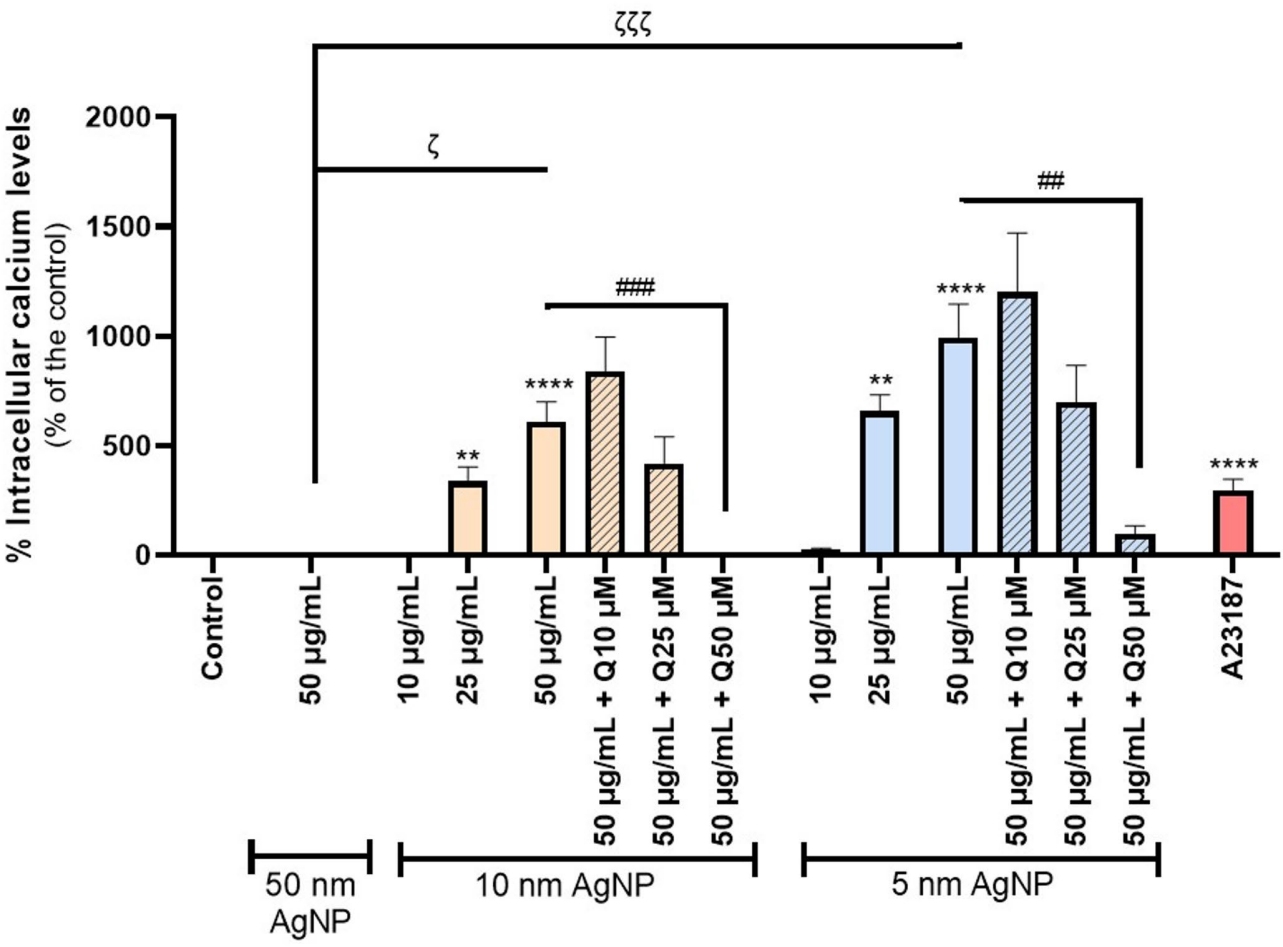
Intracellular calcium levels in erythrocytes after exposure to 50, 10 and 5 nm citrate-coated AgNP (10–50 µg/mL) and A23187 (5 µM), and the modulatory effect of quercetin (10–50 µM). **p < 0.01, ****p < 0.0001, compared to control (untreated cells), ^ζ^ *p* < 0.05, ^ζ ζ ζ^ *p* < 0.001, as indicated by the connecting lines and ^##^*p* < 0.01, ^###^*p* < 0.001, compared to citrate-coated AgNP, respectively. Each value represents the mean ± SEM of at least three independent assays

The results presented in the Fig. 6 demonstrate significant increase in intracellular calcium levels following exposure to citrate-coated AgNP, particularly with the smaller ones (5 and 10 nm). Interestingly, even at a lower concentration (25 µg/mL), 5 and 10 nm citrate-coated AgNP induced a marked elevation in calcium levels. In contrast, exposure to 50 µg/mL of 50 nm citrate-coated AgNP made no meaningful change on calcium levels. The involvement of PKC in AgNP-induced calcium influx was also investigated using a selective PKC inhibitor on 5 and 10 nm citrate-coated AgNP (only the highest concentration 50 µg/mL tested). No significant changes were observed (data not shown).

Subsequently, the protective potential of quercetin (10, 25, and 50 μM) against citrate-coated AgNP-induced intracellular calcium elevation was evaluated using the smallest NP sizes (5 and 10 nm) at 50 μg/mL. Quercetin significantly prevents the increase in calcium levels, thereby demonstrating its capacity to restore intracellular calcium levels to near control levels. However, this protective effect was observed only at the highest quercetin concentration (50 µM). Additionally, neither Ag^+^, citrate nor quercetin per se induced any significant changes in the intracellular calcium levels (Fig. S5). The A23187 (5 µM) was used as a positive control.

## Discussion

The present study pursued two main goals: the first was to evaluate and confirm the effects of AgNP on human erythrocytes, and the second was to assess the potential protective role of quercetin against AgNP-induced damage. It is essential to note that the highest AgNP concentration employed in this study (50 µg/mL) was chosen based on previous exposure studies (Freitas et al. 2020; Gliga et al. 2014). One study conducted in an AgNP production facility reported a cellular accumulation of approximately 10 µg/mL after several weeks of occupational exposure, highlighting the potential for bioaccumulation in workers chronically exposed to AgNP and underscoring the importance of evaluating long-term health risks (Gliga et al. 2014). However, overall accumulation could be even higher, since AgNP exposure can also occur outside occupational settings. Individuals may also be chronically exposed through the widespread use of AgNP in consumer products. In fact, it has been estimated that daily ingestion of AgNP ranges from approximately 20 to 80 µg per person, suggesting that dietary intake could substantially contribute to the overall body burden of AgNP (De Matteis 2017). In line with the second objective of this study, we evaluated the potential protective effect of quercetin when administered prior to exposure to AgNP. Quercetin was selected due to its well-documented antioxidant properties, as well as its natural occurrence in commonly consumed foods within the human diet (Aghababaei and Hadidi 2023; Carrillo-Martinez et al. 2024). Additionally, quercetin has been shown to demonstrate a protective effect against the deleterious effects of AgNP in other cellular models/organisms (Elblehi et al. 2022; Goodarzi et al. 2023; Martirosyan et al. 2014, 2016; Rufino et al. 2021; Sousa et al. 2025a). Although the concentrations of quercetin typically achieved through diet and plasma bioavailability are typically low, it is plausible that comparable levels could be attained through dietary supplementation (Zbikowska et al. 2014).

To the best of the current knowledge, this is the first study to evaluate the effects of three sizes of citrate-coated AgNP (5, 10, and 50 nm) on human erythrocytes, revealing their ability to trigger key events related to eryptosis and the first report to assess the protective action of quercetin against AgNP-induced toxicity on erythrocytes. In addition to evaluating AgNP-induced effects and the impact of quercetin pre-treatment, this study also represents the first report assessing the effects of citrate (coating) alone, for which no significant alterations were observed in any of the evaluated parameters. Moreover, a similar lack of effect was also noted for Ag^+^ and quercetin when tested alone.

TEM characterisation of the 5, 10, and 50 nm citrate-coated AgNP confirmed that the observed particle sizes were consistent with the expected nominal values. Consistently, when analysed in a different medium (RPMI 1640), the TEM results showed no alteration in the physicochemical characteristics or particle size, with AgNP dimensions remaining essentially unchanged from those reported by the manufacturer (Sousa et al. 2023).

As an initial step, this study evaluated the ability of citrate-coated AgNP to cause haemolysis, a key parameter commonly used to assess cytotoxicity in erythrocytes (Sæbø et al. 2023). The results demonstrated that citrate-coated AgNP of three different sizes induced haemolysis in a size-dependent manner. Notably, after 5 h of exposure, a significant haemolysis was observed for all sizes of citrate-coated AgNP. Even so, the 10 and 50 nm citrate-coated AgNP showed a significant effect only at the highest concentration tested (50 µg/mL), while the 5 nm citrate-coated AgNP induced a significant response even at the lower concentration of 25 µg/mL. Consistent with our data, Ferdous et al. (2018) reported that exposure of mouse erythrocytes to 10 nm citrate-coated AgNP at concentrations of 2.5, 10.0, and 40.0 µg/mL induced haemolysis at a concentration of 40.0 µg/mL. Bian et al. (2019) also investigated the haemolytic potential of AgNP (10 to 500 µg/mL) with an average diameter of 49.3 nm in human erythrocytes after 4 h of exposure. A significant haemolysis was observed only at the highest tested concentrations (250 and 500 µg/mL). Our results showed that the smallest citrate-coated AgNP (5 and 10 nm) induced a higher haemolytic effect, compared to the largest AgNP (50 nm). In line with these findings, Chen et al. (2015),   evaluated the effects of AgNP of three sizes (15, 50, and 100 nm), at concentrations ranging from 1.25 to 20 μg/mL, on fish erythrocytes after 2 h of exposure. The author reported that the 15 nm AgNP induced haemolysis, exceeding 60% at the highest concentration, while the 50 and 100 nm AgNP caused less than 12%. These findings, even across different species and experimental conditions, consistently demonstrate a size-dependent effect on haemolysis induced by AgNP, reinforcing the results observed in this work. Chen et al. (2015) also demonstrate in fish erythrocytes (*Carassius auratus*) using TEM analysis, that smaller AgNP were more easily internalised compared to the larger AgNP.

We also evaluated the influence of quercetin when added prior to the erythrocytes exposure to citrate-coated AgNP. The results demonstrated that quercetin significantly prevented haemolysis induced by all the citrate-coated AgNP tested. These findings suggest that quercetin can confer a protective effect against AgNP-induced membrane damage in erythrocytes. Nonetheless, other studies using erythrocytes have also demonstrated quercetin’s ability to prevent haemolysis in several contexts. In a study conducted by Manikanta et al. (2025), human erythrocytes were exposed to cell-free histones at a concentration of 100 µg/mL and simultaneously with quercetin (10–100 µM). The authors concluded that quercetin effectively delayed histone-induced haemolysis at all tested concentrations. In another study, Das et al. (2013) used erythrocytes from Swiss albino mice treated with quercetin (100 mg/kg body weight) for three consecutive days prior to gamma irradiation. The authors observed that quercetin administration resulted in a significant reduction in irradiation-induced haemolysis, possibly due to its ability to chelate free iron, which may be released during the oxidative breakdown of haemoglobin. By binding iron, quercetin can reduce the likelihood of initiating the Fenton reaction, thereby limiting further oxidative damage.

To gain further insight into the mechanisms underlying citrate-coated AgNP-induced damage and subsequent erythrocyte death, some OS biomarkers, specifically RS generation and total GSH levels, were evaluated. The results showed that all the tested citrate-coated AgNP increased the RS levels, demonstrating a concentration and size-dependent effect. Bian et al. (2019) exposed human erythrocytes to 49.3 nm AgNP, for 4 h, at concentrations ranging from 10 to 100 µg/mL. A significant increase in RS production was observed even at 50 µg/mL. Moreover, in an in vivo study, Shrivastava et al. (2014) administered 20 nm AgNP to male Swiss albino mice at doses of 1–2 µM/kg for 14 days. After this treatment, erythrocytes were isolated, and RS levels were measured. Their results showed that even the lowest dose was sufficient to induce a significant oxidative response, evidenced by an increase in RS. These studies support our findings by demonstrating that AgNP can enhance RS generation across different species, NP sizes, or exposure conditions. The consistency of these results highlights the inherent oxidative potential of AgNP that could potentially arise from both surface-mediated redox reactions or secondary intracellular oxidative responses. These findings underscore the relevance of RS production as a possible mechanism of AgNP-induced toxicity in erythrocytes. In the present work, a size-dependent effect was also observed in the generation of RS, with the smaller citrate-coated AgNP (5 nm) inducing significantly higher RS levels compared to the 10 and 50 nm AgNP. Although studies investigating the effects of different AgNP sizes on erythrocytes are limited, research on other cell lines has also demonstrated that NP size can significantly influence their biological impact, showing that smaller AgNP induce more damage (Carlson et al. 2008).

Additionally, the pre-incubation with quercetin effectively prevented the AgNP-induced increase in RS, even at low concentrations. Specifically, quercetin counteracted RS generation at concentrations as low as 0.5 µM in the case of 10 and 50 nm AgNP, and at 1 µM in the presence of 5 nm AgNP. These findings are particularly promising, considering that such concentrations can realistically be achieved through diet (Mannen et al. 2019). The antioxidant effect of quercetin in erythrocytes has also been demonstrated in a study conducted by Remigante and colleagues (2022), in which human erythrocytes were exposed to 20 mM H_2_O_2_, and the protective potential of 10 µM quercetin added before or after exposure was evaluated. The authors showed that quercetin was able to prevent the increase in reactive oxygen species (ROS) induced by H_2_O_2_. Similarly, a study conducted by Das et al. (2013) demonstrated that erythrocytes from Swiss albino mice treated with quercetin (100 mg/kg body weight), for 3 consecutive days prior to gamma irradiation, exhibited reduced levels of RS. This protective effect may be attributed to quercetin well-documented ability to scavenge RS (Carrillo-Martinez et al. 2024; Zou et al. 2021), thereby reducing oxidative damage.

Subsequently, total GSH levels were assessed as a second biomarker of redox homeostasis. GSH is the most common intracellular antioxidant, being found in high concentrations in red cells. The results obtained herein showed that 10 and 50 nm citrate-coated AgNP significantly reduced total GSH levels. Interestingly, the smallest AgNP (5 nm) did not produce a significant alteration in total GSH levels, although a decreasing trend was observed. In a previous study, Massarsky et al. (2014) exposed erythrocytes from hatchery-reared female rainbow trout (*O. mykiss*,* Salmonidae*) to AgNP with an average diameter of 9 nm (stabilized with sodium polyacrylate) at concentrations corresponding to total silver from 3.1 to 31.0 µg/mL for 48 h. Their results in erythrocytes from hatchery-reared female rainbow trout showed a significant decrease in total GSH levels, supporting the findings of the present study despite differences in the species, NP coating, and time of exposure. In addition to total GSH, several studies have focused on evaluating reduced GSH (Bian et al. 2019; Ferdous et al. 2018). Ferdous et al. (2018) reported that 10 nm citrate-coated AgNP caused a significant reduction in GSH in mouse erythrocytes at concentrations of 2.5, 10.0, and 40.0 µg/mL. Similarly, Bian et al. (2019) observed a significant decrease in GSH levels in human erythrocytes exposed to 49.3 nm AgNP, at concentrations ranging from 10 to 100 µg/mL, after 4 h of exposure. Overall, these results highlight the decrease in total GSH as an important mechanism mediating AgNP-induced toxicity in erythrocytes. Despite this oxidative-related impact, quercetin did not exhibit a significant protective effect on the levels of the antioxidant molecule.

Energy depletion is a well-established trigger of mechanisms involved in the initiation of eryptosis, as it disrupts cellular homeostasis (Pretorius et al. 2016; Tkachenko et al. 2025). In this context, to investigate whether citrate-coated AgNP affect erythrocyte energetic integrity, ATP levels were determined following AgNP exposure. In the present study, exposure to all three sizes of AgNP, at the highest concentration tested, resulted in a significant decrease in ATP levels. These findings were corroborated by Bian et al. (2019), who exposed human erythrocytes to 49.3 nm AgNP for 4 h, at concentrations ranging from 10 to 100 µg/mL and reported a significant decrease in ATP levels, which was evident even at 50 µg/mL. Interestingly, pre-treatment with quercetin led to an even greater reduction in ATP levels. A possible mechanistic explanation for this effect could lie in quercetin ability to activate certain ATPases, particularly Na⁺,K⁺-ATPase. These enzymes play a critical role in maintaining the ionic gradient across the plasma membrane by actively transporting 3Na⁺ ions out of the cell and 2 K⁺ ions into the cell, a process that is energetically costly and requires the hydrolysis of ATP (Kumar and Maurya 2021). For instance, a study conducted by Kumar et al. (2021) in human erythrocytes from donors of different age groups, shown that quercetin enhance Na⁺,K⁺‑ATPase activity, even at very low concentrations (ranging from 10^− 6^ to 10^− 3^ M), highlighting its impact in energy-consuming ion transport processes. Furthermore, Manikanta et al. (2025) demonstrated that in human erythrocytes exposed to cell-free histones (100 µg/mL) and simultaneously to quercetin (25–50 µM), this flavonoid showed the ability to significantly increase Na⁺,K^+^-ATPase activity affected by the cell-free histones. Since this enzyme directly consumes ATP, its activation by quercetin may explain the reduction in ATP observed. This suggests that in erythrocytes exposed to AgNP, the presence of quercetin might stimulate Na⁺,K⁺‑ATPase activity as part of a compensatory response to restore ionic homeostasis.

It is currently accepted that OS and energy depletion act as major stimuli for eryptosis by promoting an increase of intracellular calcium levels. Since mature erythrocytes lack mitochondria and the conventional apoptotic machinery, alterations in calcium homeostasis represent a critical signalling mechanism driving the activation of eryptotic pathways. (Föller and Lang 2020; Pretorius et al. 2016; Tkachenko et al. 2025). For this reason, intracellular calcium levels were assessed in the present study. Our results demonstrated that exposure to 5 and 10 nm citrate-coated AgNP led to a significant increase in intracellular calcium levels. Similar results were obtained in the study conducted by Ferdous et al. (2018), which showed a significant increase in intracellular calcium levels, even at the lowest AgNP concentration tested (2.5 µg/mL). Interestingly, although the 50 nm AgNP induce an increase of RS and a decrease in ATP and GSH levels, no corresponding increase in intracellular calcium levels was detected. This observation may further reinforce that the size of the AgNP plays a critical role in determining the mechanism of toxicity, with smaller AgNP potentially triggering calcium-mediated eryptotic pathways, whereas larger particles may act through alternative mechanisms. Nevertheless, this discrepancy may be explained by limitations in the sensitivity of the method employed or alternatively, despite the presence of OS and ATP depletion, the extent of these effects may have been insufficient to initiate the processes leading to calcium influx.. A study conducted by Bian et al. (2019) reported a significant increase in intracellular calcium levels in erythrocytes, exposed to 49.3 nm AgNP, even at a concentration of 50 μg/mL. These results diverge from those of the present study, in which 50 nm citrate-coated AgNP did not induce any changes in intracellular calcium levels. This discrepancy may reflect differences in experimental conditions, particularly the use of a different detection technique, longer incubation periods, and a higher probe concentration.

Interestingly, the prior treatment of quercetin demonstrated a calcium-lowering effect. This aligns with findings from Manikanta et al. (2025), who reported that human erythrocytes exposed to cell-free histones (150 µg/mL) exhibited a pronounced increase in intracellular calcium, which was effectively mitigated by quercetin at concentrations between 25 and 50 µM. These results support the hypothesis that quercetin possesses a protective effect against calcium overload, likely due to its antioxidant properties. By scavenging RS, quercetin may suppress the activation of calcium-permeable channels, thereby preventing excessive intracellular calcium accumulation. The possible involvement of 5 and 10 nm AgNP in the PKC-mediated calcium signalling pathway was further investigated. However, no significant effects were detected, showing that PKC is not directly involved in AgNP-induced calcium dysregulation.

## Conclusions

In conclusion, all three sizes of citrate-coated AgNP (5, 10, and 50 nm) were found to induce damage to human erythrocytes, including haemolysis, RS formation and total GSH decrease, ATP depletion, and dysregulation of intracellular calcium levels. These findings highlight the capacity of AgNP to trigger critical processes associated with eryptosis in human erythrocytes. Notably, particle size emerged as a determinant of toxicity, with smaller AgNP, particularly the 5 nm AgNP, eliciting the strongest adverse responses. This size-dependent effect may be attributed either to the increased surface reactivity and/or the higher particle number inherent to smaller NP at equivalent µg/mL concentrations. These combined factors may contribute to the enhanced toxic responses observed. In contrast, no significant effects were observed for Ag⁺ or citrate alone, suggesting that the observed harmful effects are specifically related to the NP form.

Quercetin demonstrated a clear protective effect against several major harmful outcomes induced by AgNP, particularly by reducing haemolysis, RS production, and intracellular calcium elevation. However, quercetin did not show any effect on total GSH levels, and notably, its presence was associated with a further decrease in ATP.

Overall, these findings underscore the cytotoxic potential of AgNP and the protective role of quercetin, emphasizing the urgent need to define safe exposure limits and to explore flavonoids as potential modulators of NP-induced toxicity.

## Supplementary Information

Below is the link to the electronic supplementary material.

Supplementary Material 1
