# Supplementary material for: Quercetin mitigates size-dependent oxidative and metabolic toxicity of citrate-coated silver nanoparticles in human erythrocytes
Source: Arch Toxicol. 2026 Feb 17;100(5):1837–50. doi: 10.1007/s00204-026-04308-z (PMC13086775; doi:10.1007/s00204-026-04308-z)
Supplement: Supplementary file 1 — Supplementary Material 1 [file 204_2026_4308_MOESM1_ESM.docx]

**Supplementary Information**

**Quercetin mitigates size-dependent oxidative and metabolic toxicity of citrate-coated silver nanoparticles in human erythrocytes**

**Inês Santos^1^, Vera M. Costa^2,3,4^, Félix Carvalho^2,3^, Eduarda Fernandes^1*^, Marisa Freitas^1*^**

^1^LAQV, REQUIMTE, Laboratory of Applied Chemistry, Department of Chemical Sciences, Faculty of Pharmacy, University of Porto, 4050-313 Porto, Portugal

^2^UCIBIO ‑ Applied Molecular Biosciences Unit, Laboratory of Toxicology, Department of Biological Sciences, Faculty of Pharmacy, University of Porto, 4050‐313 Porto, Portugal

^3^Associate Laboratory i4HB ‑ Institute for Health and Bioeconomy, Faculty of Pharmacy, University of Porto, 4050‑313 Porto, Portugal

^4^ Faculty of Medicine, University of Porto (FMUP), Rua Alameda Prof Hernâni Monteiro, 4200-319, Porto, Portugal

* Correspondence: [marisafreitas@ff.up.pt](mailto:marisafreitas@ff.up.pt) / [egracas@ff.up.pt](mailto:egracas@ff.up.pt)

Journal name: Archives of Toxicology


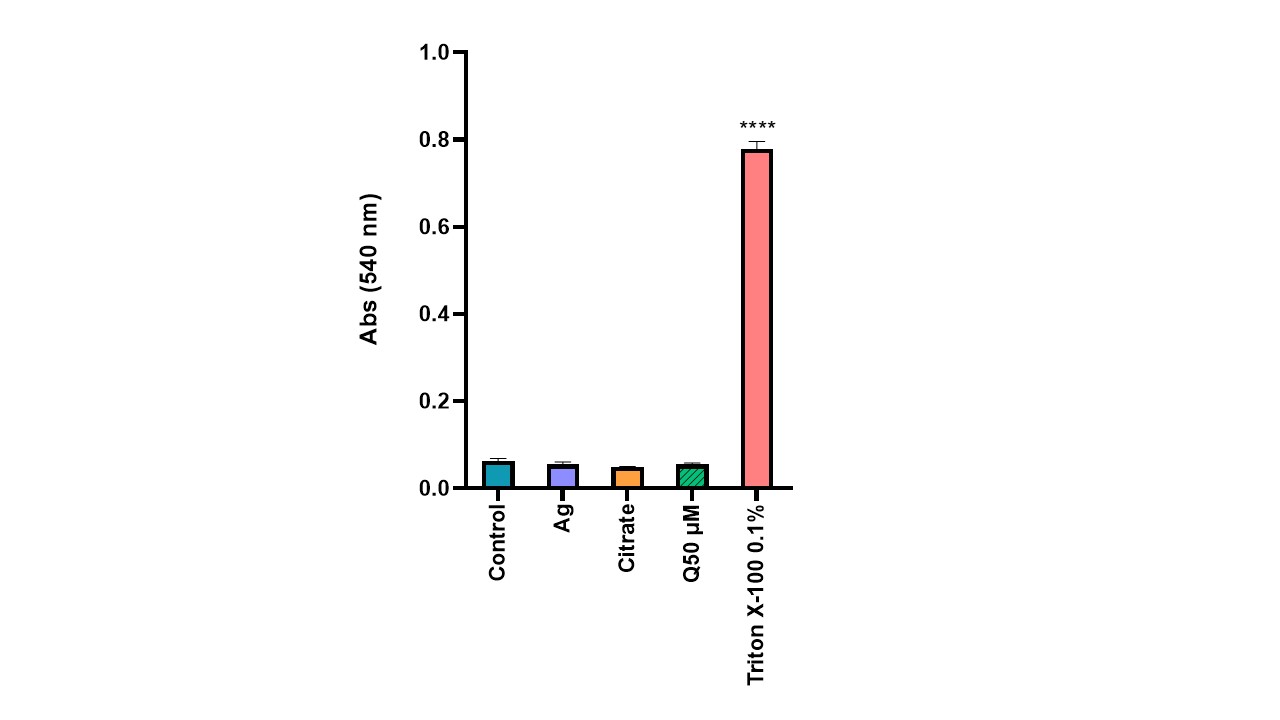


**Fig. S1** Haemolytic effect to Triton X-100 (0.1%), Ag^+^ (0.5 µg/mL), citrate (0.02 µg/mL) and quercetin (50 µM) *per se* on erythrocytes after 5 hours of exposure. ****p<0.0001, compared to control (untreated cells). Each value represents the mean ± SEM of at least three independent assays.


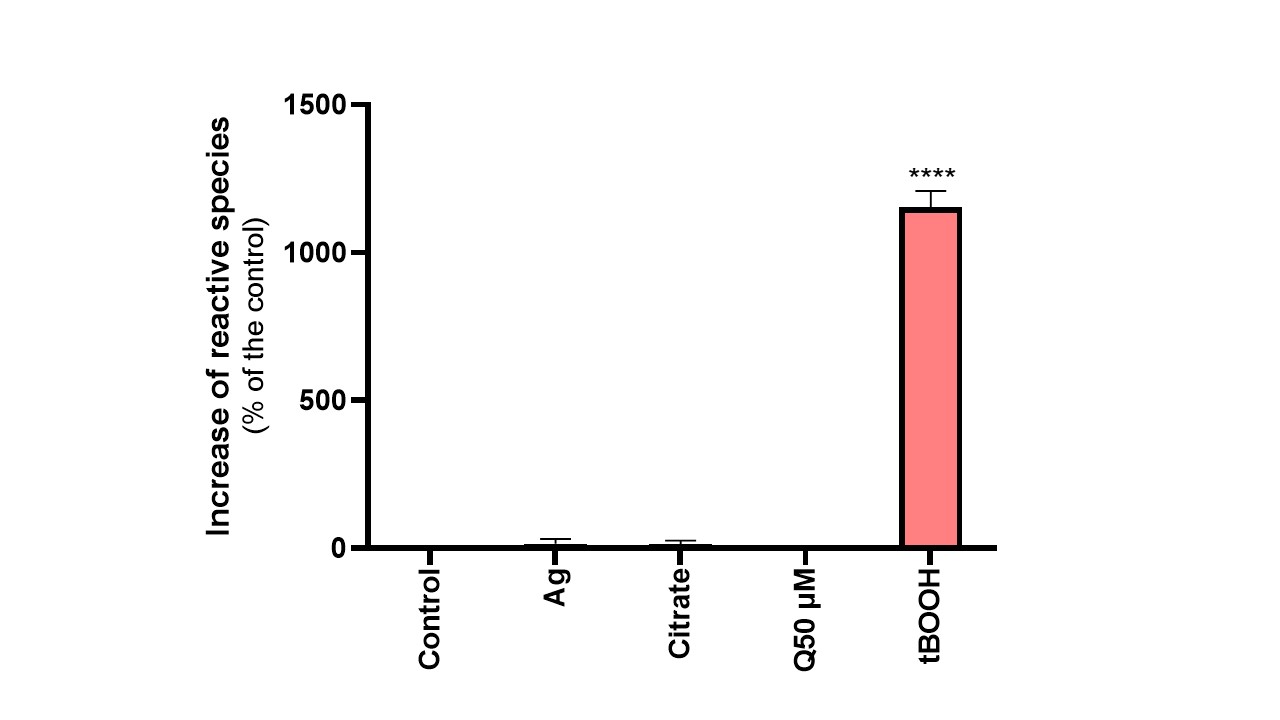


**Fig. S2** Reactive species (RS) formation by erythrocytes to tBOOH (20 µM), Ag+ (0.5 µg/mL), citrate (0.02 µg/mL) and quercetin (50 µM) per se. ****p<0.0001, compared to control (untreated cells). Each value represents the mean ± SEM of at least three independent assays.


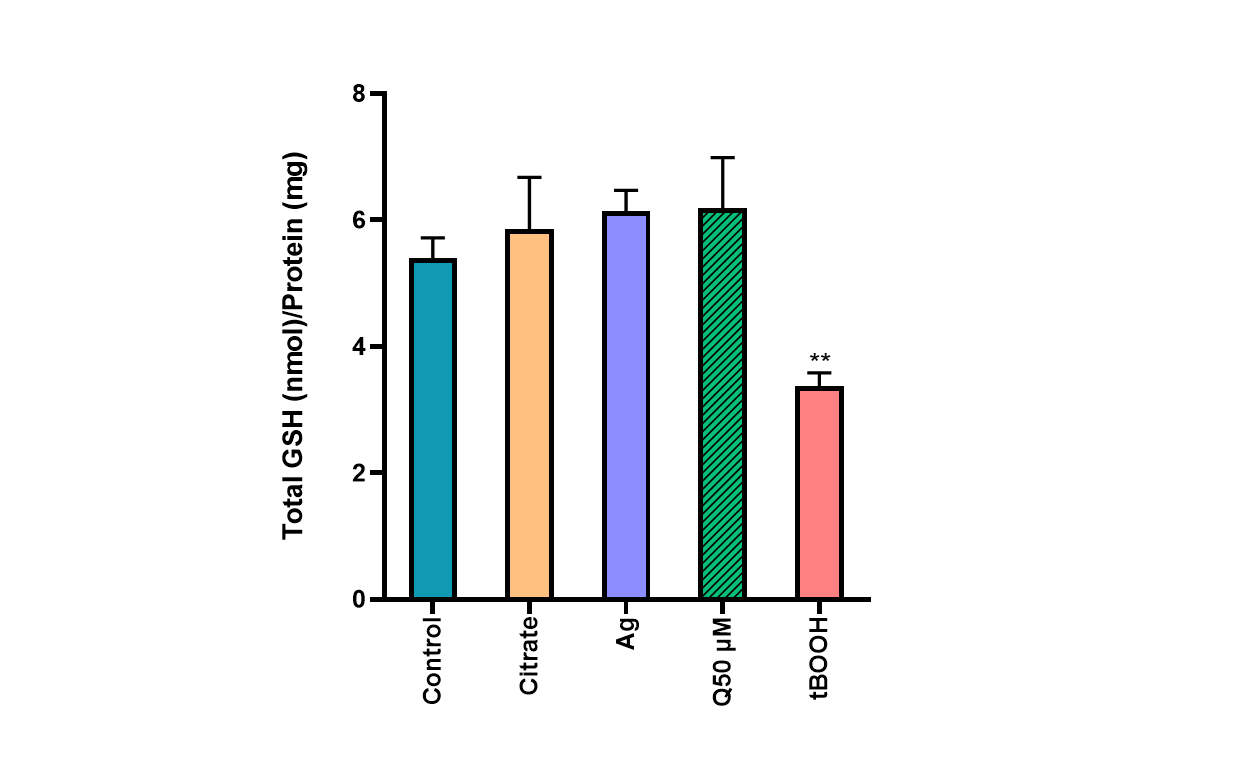


**Fig. S3** Total GSH levels on erythrocytes to tBOOH (250 µM), Ag^+^ (0.5 µg/mL), citrate (0.02 µg/mL) and quercetin (50 µM) *per se*. **p<0.01, compared to control (untreated cells). Each value represents the mean ± SEM of at least three independent assays.


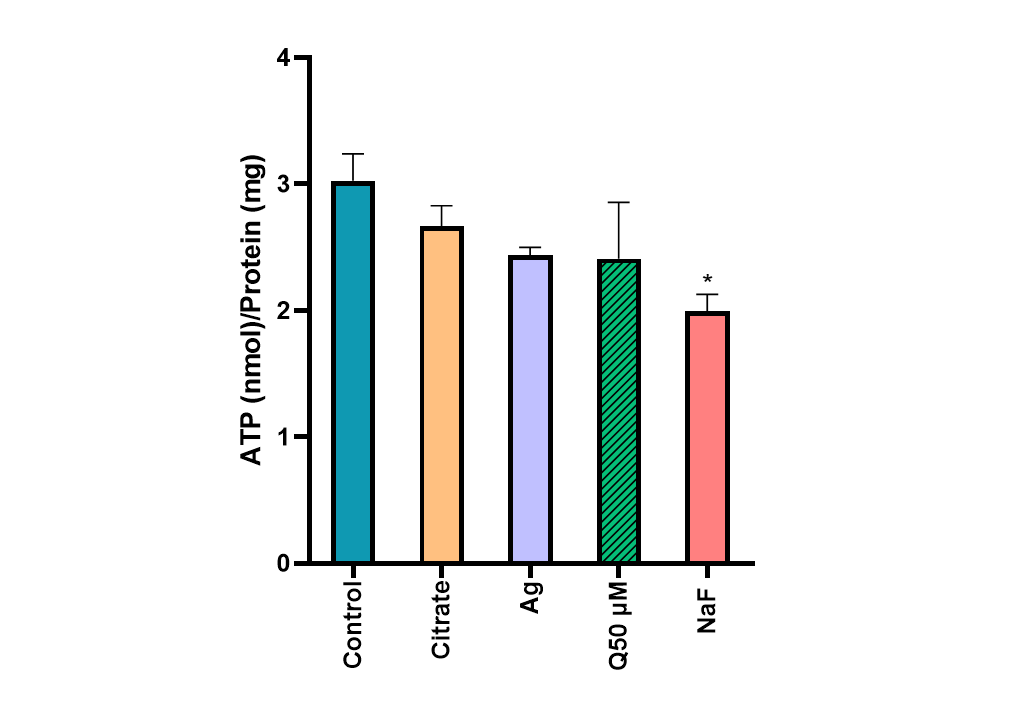


**Fig. S4** ATP levels on erythrocytes after exposure to NaF (200 µM), Ag^+^ (0.5 µg/mL), citrate (0.02 µg/mL) and quercetin (50 µM) *per se*. *p<0.05, compared to control (untreated cells). Each value represents the mean ± SEM of at least three independent assays.


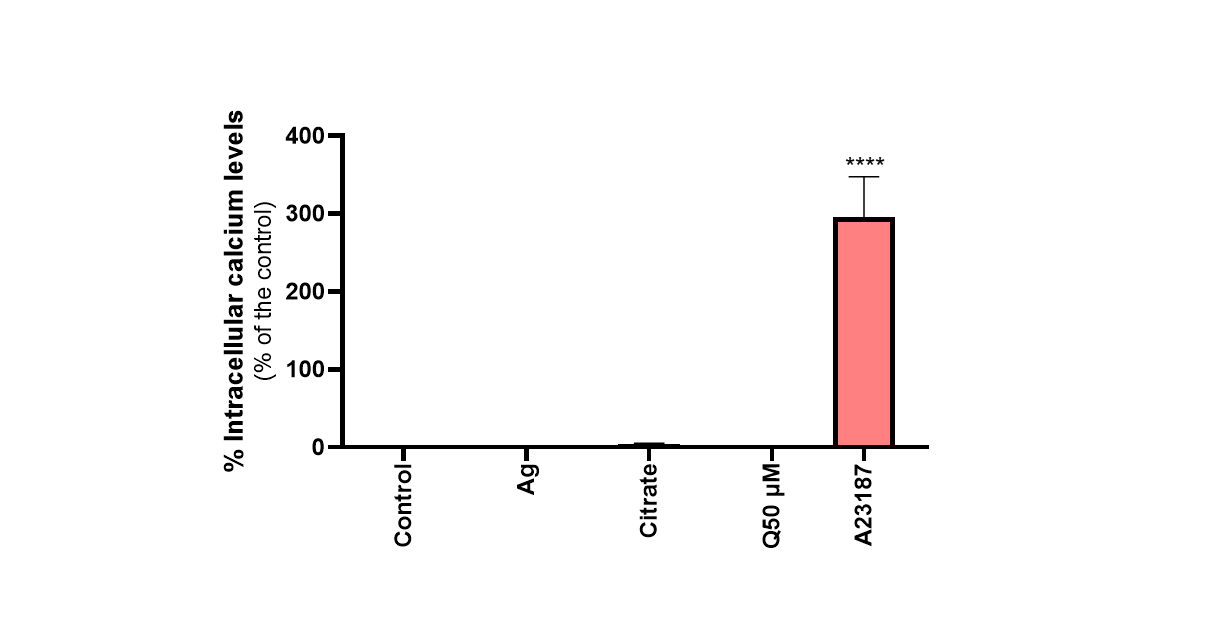


**Fig. S5** Intracellular calcium levels on erythrocytes after exposure to A23187 (5 µM), Ag^+^ (0.5 µg/mL), citrate (0.02 µg/mL) and quercetin (50 µM) *per se*. ****p<0.0001, compared to control (untreated cells). Each value represents the mean ± SEM of at least three independent assays.
